# The Reasons for Early Weaning, Perceived Insufficient Breast Milk, and Maternal Dissatisfaction: Comparative Studies in Two Belgian Regions

**DOI:** 10.1155/2014/678564

**Published:** 2014-11-09

**Authors:** Emmanuelle Robert, Yves Coppieters, Béatrice Swennen, Michéle Dramaix

**Affiliations:** ^1^Research Center of Epidemiology, Biostatistics and Clinical Research, School of Public Health, Université Libre de Bruxelles, Route de Lennik 808, 1070 Brussels, Belgium; ^2^Research Center of Health Policy and Systems-International Health, School of Public Health, Université Libre de Bruxelles, Route de Lennik 808, 1070 Brussels, Belgium

## Abstract

*Objective and Method*. To report on the weaning reasons at the maternity ward, at 3, 6, and 12 months and to report the socioeconomic characteristics of mothers not satisfied with breastfeeding duration as well as of those who have weaned their child because of perceived insufficient milk (PIM). Two cross-sectional studies were performed in 2012. *Results*. 62.9% of mothers in Wallonia and 56.8% in Brussels are dissatisfied with the duration of breastfeeding. In the two regions, younger mothers, ignoring the WHO recommendations, having a low level of education, or thinking not having sufficient milk production, were more likely to be dissatisfied. According to the analysed period, PIM and return to work are the two leading causes of weaning. While in Brussels PIM seemed to be associated only with partial BF at the maternity ward, in Wallonia, PIM was associated with a less educated environment and with ignoring the WHO recommendations. *Conclusions*. Too many mothers, especially destitute, are dissatisfied. They more often evoke PIM as reason for weaning. However, the literature shows that the real lack of milk only affects 1–5% of the mothers. Professionals need to be better informed of this discrepancy between mothers' perception and physiology. They should be more supportive, especially among more precarious mothers.

## 1. Introduction

The obvious benefits of breastfeeding (BF) for both the child and the mother are fully described [1]. In order that many children can benefit from these advantages, the World Health Organization (WHO) recommends exclusive BF since 2001 for a period of 6 months and supplemented breastfeeding for at least two years [2]. Despite the fact that many countries have included these recommendations in the guidelines of various paediatric societies, very few mothers achieve these two objectives in many countries in Europe [3], in the United States [4, 5], or in other parts of the world [3, 6, 7].

Even if social-demographic predictors of both the initiation [8–10] and the duration of BF are widely described [9, 11–13], very few studies consider at the same time (i) the mothers' desired BF duration, (ii) the dissatisfaction generated by not achieving a certain BF duration, and (iii) the primary reason for weaning. It seems yet obvious that breastfeeding is a personal choice and the mothers' desired duration cannot be ignored.

These elements could partially explain why the initiation rates and also the BF duration remain too low according to the WHO recommendations on which, however, the vast majority of scientists agree.

The literature shows that the reasons for an “early” weaning, defined as not achieving the mother's desired breastfeeding duration, depend on various factors including psychosocial (self-esteem, self-efficacy), cultural factors [6], but also the duration of the maternity leave [14, 15]. The perception of insufficient milk (PIM), that is, the mother's belief that the breast milk is inadequate in amount or nutritional quality to meet her infant's needs [16], is another factor often highlighted [4, 17]. This phenomenon seems to exist in many countries, without cultural neither social-demographic borders [4]. The introduction of formula supplementation in the maternity ward is also a main early weaning factor [18].

Two representative studies on BF were simultaneously performed in Wallonia and in the Brussels-Capital Region, two of the three regions that form Belgium. Analyses regarding the durations of any and exclusive breastfeeding in Wallonia were previously described [13]. Specifically in this paper, we will try to answer different questions about early weaning. Is there a link between the desired and the achieved duration? What is the profile of the mothers not satisfied with their breastfeeding duration? What is the distribution of the principal reasons of weaning at different moments during the first year postpartum? What are the predominant reasons for weaning? What are the social-economic factors associated with PIM? And finally are these results different between the two regions?

## 2. Method

### 2.1. Population and Samplings

In Wallonia, a regional immunization survey was performed every 3 years and approximately every 5 years in the Brussels-Capital Region (Brussels). In 2012, the two surveys were performed in the same time, with the same questionnaire. For the first time, a set of 16 questions about breastfeeding was introduced in these two vaccination coverage surveys that took place from May to July. More details for the methodology were included in the previous articles about vaccination [19] and breastfeeding [13]. Using Epi-methodology, we firstly selected in Wallonia a proportionate sample of 55 clusters in 50 municipalities (larger municipalities to be drawn more than once). In the second stage, 12 children per cluster were randomly selected from the municipalities list.

In Brussels, a stratified sampling with allocation proportional to size of the municipality was used in each of the 19 municipalities of Brussels.

A list of children born between 31 of May and 30 of November 2010 in the two studies was obtained from each municipality. All children were officially registered as residents in Brussels or in Wallonia. The families were informed by letter that an interviewer would visit them regarding a survey on “infancy.” If the families could not be contacted after 3 visits, had moved, or had serious language problem they were replaced. If necessary, information was cross-checked via phone calls to the parents, after reviewing every questionnaire.

Both databases were registered by the Commission for the protection of the privacy in Belgium.

### 2.2. Measurements

For the definition of “exclusive breastfeeding” we used the WHO definition: the intake of breast milk (directly, expressed or from a wet nurse) without any additional liquids or solid/semisolid foods; intakes of oral rehydration solution (ORS), vitamins, minerals, or medications in the form of drops or syrups are allowed [2].

We classified the 90 reasons provided by the parents for no breastfeeding, no exclusive BF, or weaning into 15 major reasons. These 15 reasons were classified into 2 groups (intrinsic BF problems and external causes), divided into subcategories.

Intrinsic BF problems were considered as direct causes linked with BF problem. A difference was made between PIM and no/no more milk. The perception of insufficient milk (PIM) is defined as a mother's belief that her breast milk is inadequate in amount or nutritional quality to meet her infant's need [16]. No/no more milk is less subjective than PIM. The mother observed she had no milk.

External causes were considered as not directly linked with a BF problem.  Table 1 shows nonexhaustive examples belonging to each class.

The reasons of non-BF and partial BF were analysed at the maternity ward and the reasons of weaning were analysed at three different moments: ≤3 months, ≤6 months, and ≤12 months.

### 2.3. Statistical Analysis

Pearson's chi square was used to compare the characteristics of the two regions. After the description of the two samples, only mothers who breastfed at the maternity ward were included in the analyses. The duration of BF was calculated considering the month in which BF was stopped. The median durations and 95% CI of any and exclusive BF were derived using Kaplan-Meier survival curves. The log-rank test was used to assess the equality of the survival curves.

We examined the relationship between the mother's dissatisfaction and different predictors (e.g., mother's native nationality and education level, mode of delivery, parity, etc.) with Pearson's chi square test. Odds rations (OR) and their 95% CI were computed. The relationship between mothers cited PIM as main reason for weaning and different predictors were assessed using Pearson's chi square test. Two cutoffs, at 5 months for exclusive BF and 6 months for any BF, are considered for PIM analyses. These limits allow having a sufficient number of children regarding each predictor in both regions. For all analyses mentioned above, the significance level was 0.05. A logistic model was used to analyse the predictors associated with dissatisfaction in each region. The covariates included in the models were selected by a backward stepwise procedure. All variables associated with the dissatisfaction of BF duration with *P* values ≤ 0.10 were included in the final model. Goodness of fit was checked using Hosmer-Lemeshow's test. We did not pool the two databases because of the large difference in sociodemographic characteristics between the two populations. Epi-Info 6.04d Fr (Centre for Disease Control and Prevention) was used for encoding and analyses were performed with IBM SPSS 22.0.

## 3. Results

The data concerned 525 children in Wallonia and 544 children in Brussels. Sociodemographic characteristics of parents and children and the prevalence of BF (exclusive, partial, and no BF) at birth and at discharge from the maternity unit and its duration are shown in Table 2. Almost all these parameters were significantly different between the two regions. A greater number of parents were little educated in Brussels and had a lower income. The mothers were older, more often of foreign origin, and worked less often than in Wallonia. More children were born in a “Baby Friendly Hospital Initiative” (BFHI) maternity in Brussels. The prevalence of breastfeeding was by more than 10% higher in Brussels. This difference was the same for exclusive BF, any breastfeeding at birth or at discharge of maternity ward. BF durations were longer in the capital. The proportion of children partially breastfed at discharge from the maternity unit was on the contrary the same in both regions. More mothers took the decision to breastfeed before pregnancy and did not know the WHO recommendations in Brussels. A larger number of fathers were not in favour of BF in Wallonia (Table 2).

### 3.1. Desired Duration of Any Breastfeeding and Median Breastfeeding Duration Performed

Mothers had higher duration objectives in Brussels, where over 53% of them wanted to breastfeed for one year at least. This result was 22% in Wallonia. In contrast, in Wallonia more mothers wanted to breastfeed for 6 months maximum. It was the same for mothers who had no clear idea regarding the desired length.

In the two regions, the median durations of any breastfeeding were longer if mothers had greater desires in BF duration (*P* < 0.001). The median durations were always shorter than the desired durations.

A gradient between desired and performed duration of BF in the two regions was observed. In Brussels, not wishing a particular duration was favourable to the duration achieved (Table 3).

### 3.2. Characteristics of Mothers Who Could Not Achieve Their Breastfeeding Duration Objectives

In Wallonia, among 429 mothers who initiated BF at birth, 62.9% (253) were not satisfied with its length. In Brussels, among 506 mothers who initiated BF at birth, 56.8% (273) were not satisfied (*P* = 0.06).  Table 4 shows the percentage of dissatisfaction with the characteristics of these dissatisfied mothers in both regions.

In both regions, the youngest mothers, mothers who did not know the WHO recommendation, those who did not have a higher level of education, mothers who are perceived to have less milk (PIM), and those who had a longer period breastfeeding desire were more likely to be dissatisfied with any BF duration.

In Wallonia, the mothers of a first child, a child born prematurely, or those who had returned to work a maximum of 3 months after birth had a greater likelihood of not achieving their BF duration objective.

In Brussels, a lot of mothers cannot reach their objective when the child was born during fall, in a nonlabelled BFHI (*P* = 0.07), when the father was not highly educated, or when the household income was lower (*P* = 0.06).

After adjustment for all significant factors <0.10 in bivariate analysis, a younger age, the ignorance of the WHO recommendation, and having had a desire to breastfeed for a certain duration (especially for more than 12 months) remained associated with a higher frequency of dissatisfaction in the two regions regarding the duration of AM achieved. Prematurity and returning to work at 3 months or sooner postpartum were also associated with dissatisfaction but only in Wallonia. In Brussels, not being born in a Baby Friendly Hospital Initiative maternity and having a perception of lack of milk were associated with dissatisfaction regarding the duration achieved.

### 3.3. Main Reason for Weaning

#### 3.3.1. At the Maternity Ward

In both Brussels and Wallonia, at the maternity ward, the “personal opinion” was the most often given reason for nonbreastfeeding (Table 5). In contrast, the second most often given reason changed by region. In Wallonia, “mother's health” or “bad previous experiences” were most often mentioned (13.0%) and in Brussels the reasons were distributed more homogeneously (column 1).

Among mothers who did not breastfeed exclusively (column 2), the “perceived lack of milk” was the most often mentioned reason in the two regions. “Technical difficulties” appear as the second factor which causes partial BF at the maternity ward. In other words, when leaving the maternity ward, the majority of the mothers (>60%) gave as a reason for partial breastfeeding a “BF intrinsic problem.”

#### 3.3.2. After the Discharge of the Maternity Ward

In the first 3 months, the main reason for weaning remained linked to a “BF intrinsic problem” (>58%), the PIM being the most often cited subcategory (approximately 30%). “Back to work” became the primary reason for stopping BF beyond three months after birth (Wallonia). “BF problems” were referred by parents of one-third of the children younger than 12 months. “The child does not want to drink” increased in the three studied periods after discharge from maternity ward.

### 3.4. Perceived Insufficiency of Breast Milk and Associated Factors

Table 6 shows the relationship between the PIM and the parent's sociodemographic status and others factors.

In Brussels, the number of mothers who exclusively breastfed at 5 months was 2 times higher than in Wallonia (38.4% versus 17.7%). At 6 months, in Brussels, while more than one in two mothers was still breastfeeding, in Wallonia, it was a mother in 3. “PIM” meanwhile was identical in the two regions and this was during both considered times.

For the final weaning (before 6 months) the PIM seemed more sensitive to various characteristics analysed in Wallonia.

Indeed, a significant disparity appeared by region. First, in Brussels, PIM as a reason for stopping the exclusive BF was distributed more homogeneously. Indeed, no significant difference was found. In Wallonia, PIM was mentioned most often for children whose mothers did not know the WHO recommendations and whose parents had less education, or when household income was lower.

In both Brussels and Wallonia, PIM as a weaning reason at less than 6 months was mentioned more often among women who had less education. Only in Brussels, having breastfed her child partially and not delivering in a BHFI maternity increased the risk of weaning because of a perceived lack of milk. In Wallonia the predictors were the same for the exclusive weaning before 5 months.

## 4. Discussion

Both regions cover 45% of the Belgian population. Brussels and Wallonia have a population completely different on sociodemographic level (see Table 1). Brussels, both the Belgian and the European Capital, is distinguished by a higher birth rate than the other two Belgian Regions. The birth rate is also one of the highest in Europe. An argument could explain this rate due to the strong immigration in Brussels. Indeed, Brussels has 75% of mothers with foreign origin including mothers who are (i) European migrants from a more affluent background, who have late pregnancies, and (ii) non-European, younger women from a less affluent background and higher multiparous.

These two groups of migrant mothers breastfeed longer. A lot of studies show that BF is longer in older mothers [9, 10, 20] but also among foreign-born mothers [8, 9]. It may therefore seem logical that Brussels has a higher proportion of mothers who are “naturally” in favor of BF. This could be illustrated by the fact that over 50% of mothers in Brussels desire to breastfeed at least one year. In Wallonia, the rate reaches only 22%. There seems to be a strong relationship between desired and achieved duration of BF as Donath demonstrated [21].

It is probable that Brussels rather than Wallonia carries a deeper “BF culture.” It is difficult to evaluate the part due to the demographic characteristics in Brussels and the part due to the BF promotion initiatives. Since 2001, in Belgium, more and more maternities are BFHI accredited every year, mostly in Brussels.

### 4.1. Dissatisfaction of the Achieved BF Duration

In our two surveys more than 50% of the mothers said they did not breastfeed long enough: the median BF durations were always shorter than the desired durations. This confirms the results of Negayama et al. and Perrine et al. [6, 22].

The dissatisfaction defined as the difference between the expected and performed BF duration seems to be related to certain factors including socioeconomic vulnerability. Younger mothers and mothers who ignore the ideal duration of exclusive BF are frequently dissatisfied. A greater number of unsatisfied mothers are also found among mothers who intended to breastfeed for at least 12 months. When analysing the dissatisfaction, some predictors “classically” strongly associated with the duration and prevalence at the maternity ward disappear. It is the case with the mother's native nationality, the attitude of the partner, and the prenatal breastfeeding intention.

According to our survey, in Wallonia, 23.5% of children were born in a BHFI maternity against 68.7% in Brussels (*P* < 0.001). However, like some authors [23] but unlike others [24, 25] our regional analysis showed no statistical differences for children born in a maternity BFHI on the prevalence, duration of BF, and dissatisfaction [13]. However, BFHI guidelines have qualitative impacts in favour of breastfeeding, probably spreading beyond accredited maternities, particularly in Brussels.

### 4.2. Principal Reason for Terminating Exclusive or Any Breastfeeding

At birth, according to the region, between 32% and 38% of the mothers did not want to breastfeed for “personal reasons.” Medical reasons related to the mother's health or incompatible with BF drugs appear in less than 13% of the cases. Except these two reasons, it is probable that, with a better support at the maternity and a better knowledge, mothers could consider BF more serenely. This should particularly be the case for the “BF intrinsic problems” already occurring at the maternity, which are responsible for the introduction of breast milk substitutes in 60% of the children. These “BF intrinsic problems” remain the main reason for weaning in the first three months postpartum. This category includes the mothers who, with a better support from professionals, could exclusively breastfeed at the maternity and wean their children later.

After 3 months postpartum in the two regions, the majority of the problems were “external causes.” In this category, “back to work” was the most important reason (41.1% in Wallonia). This reason is frequently cited as the main reason of weaning after 3 months in the literature [6, 26].

### 4.3. Perceived Insufficient Milk

Depending on the period, PIM isolated is the first or the second reason cited for initiating supplementation or weaning. This finding is consistent with previous research in many populations across the world [6, 7, 16, 27]. Across the literature, most researchers found that approximately 35% of all women wean their children early, reporting that PIM was the primary reason [4, 28]. Gatti after critical review of research pertaining to perceived insufficient milk (PIM) wrote that many women utilize infant satisfaction cues as their main indication of milk supply and many researchers, clinicians, and breastfeeding women do not evaluate actual milk supply [4]. Quintero Romero et al. in a study in Italy show that 54% mothers give an inadequacy of breast milk as reason for giving formula before six months [17]. Many authors showed that PIM was correlated with decreased exclusivity [4, 7, 29].

PIM has to be differentiated from the “primary” milk insufficiency, related to an anatomical or hormonal inability to produce milk [4, 30]. A certain amount of studies show that this “primary” insufficiency is rather rare. We tried to differentiate all the reasons that can be gathered under the definition of PIM as defined by McCarter-Spaulding and Kearney [16].

The fact that in Brussels at ≤3 months the answer “no milk at all” is chosen for 22.9% (*n* = 30) of the children is surprising. Two hypotheses could help us to understand that astonishing fact. Firstly, certain parents may think that reason is more socially acceptable. Secondly, that reason closes the debate and resists to the arguments of the people around the family. For Negayama et al. PIM is interpreted as a solution of conflict between the social pressure to breastfeed and its burden [6].

#### 4.3.1. PIM and Association with Other Variables

While in Wallonia the PIM is most frequently mentioned as a reason for weaning in the most disadvantaged populations, in Brussels this reason is more homogeneously distributed in the population. In Japan, where the BF rates are low, Otsuka et al. [7] showed PIM was not related to maternal age, parity, or income, but with mothers with higher than college education. Some researchers show an association with parity, others not. Negayama et al. have shown that mothers who reported insufficient milk were less satisfied with the duration of feeding [6]. This was also observed in the two Belgian regions.

#### 4.3.2. PIM and Supplement Breast Milk Substitute in the Maternity

Many authors have shown the negative influence of early supplement breast milk substitute in the maternity ward on the duration of breastfeeding [10, 18, 31] but also on the difficulty of achieving the target set by the mother [23]. In the 2005–2007 Infant Feeding Practices Study II in USA, after adjustment for all hospital practices, only not receiving supplement feeding remained significant for achieving exclusive BF intention [22].

The introduction of formula could be a cause rather than an outcome of the insufficiency of breast milk although it might not be the case in early period [32]. The introduction of formula reduces nipple stimulation and hence could cause a decrease in the production of breast milk [6]. In 2001, McCarter-Spaulding and Kearney wrote that the use of bottle, if accompanied by decreased BF frequency, may contribute to perceived or actual insufficient milk over time, as the infants grow and require a greater milk supply. Likewise, longitudinal observations might show that if BF problems persist, they lead to a perception of PIM and possible early weaning. We show that 37.0% of the mothers in Wallonia and 48.1% in Brussels who did not achieve exclusive BF before leaving the maternity reported that PIM was the reason.

### 4.4. Strengths and Weaknesses

Few studies combine the analyses of the weaning reasons while isolating the PIM and the lack of satisfaction regarding the duration of BF with the sociodemographic parameters and other BF parameters, differentiating the “any” and “exclusive” BF according to the WHO definitions. Moreover these analyses were about two contiguous regions but heterogeneous on a sociodemographic level, showing inside a small country two different situations.

There is still one question remaining about the knowledge of the WHO recommendations. Are the mothers more inclined to breastfeed if they know the recommendations or does knowing the recommendations have an effect on increasing the breastfeeding duration? Our data does not allow us to differentiate the cause and the effect in that case. But it is probable that motivated mothers get informed and know the target of an exclusive BF during 6 months. Study and methodological limits were described in the previous papers [19, 33].

## 5. Conclusion

In 2000, Dennis wrote “although the health benefits of BF are well documented and initiation rates have increased over the past 20 years, most mothers wean [*sic*] before the recommended 6-months [*sic*] postpartum because of perceived difficulties with BF rather than due to maternal choice” [9]. Thus, nearly 15 years later, in two of three Belgian regions this conclusion seems to be still valid. Unfortunately, we can write, as Dennis, that weaning unfortunately is often not a desired choice!

We think that the knowledge of the WHO recommendations can have a positive impact on the mothers who are less naturally inclined to breastfeed when they are aware of the ideal duration, which can be a guideline.

Because of the importance of an early BF intention, we suggest expanding more strategies to educate people at a young age, in adolescence, for example, in order to develop a “BF culture.”

The majority of weaning related to a perceived lack of milk is preventable. To do this, it is really necessary that professionals are aware of the role they can play in enhancing self-efficacy but also in the correct information they can give to parents. They should consider the logic of the law of supply and demand of milk that can be completely disrupted by unnecessary supplementation. Thus, it is important to consider the quality of information given to parents. “BF intrinsic problems” which lead to mother's dissatisfaction are early solved with an adequate support and by training the professionals of the maternity.

## Figures and Tables

**Table 1:** Classification of reasons for no breastfeeding, no exclusive breastfeeding, and weaning.

Principal group	Subcategorical	Examples of reported reasons
Intrinsic BF problems	Perceived insufficient milk (PIM)	No enough milk Poor quality of milk The child wanted to be breastfed all the time The child slept at the breast The child was crying too often The child remained all day at the breast The child was difficult to handle after BF The mother does not know whether she has enough milk
	Technical difficulties for the mother and the baby	The child is not able to drink Too difficult for the baby Crack/engorgement Mastitis
	No/no more milk	No enough milk to express and to supply the crib with No more milk

External causes	Personal opinion	Mother does not like the BF and does not want to breastfeed Too stressed to breastfeed Personal choice
	Mother's medical reason	Mother takes incompatible drugs with BF Mother's hospitalization Hepatitis C Nervous breakdown
	Circumstance of birth	Caesarean Child in an incubator, premature baby
	Baby's health	Down syndrome Harelip
	Logistics	Moving, holiday
	Other reasons	Because of the child's teeth Because of social environment Mother smoking and so forth

**Table 2:** Comparison between the two surveys (Wallonia, Brussels), *P* value.

	Wallonia	Brussels	*P* value
	% (*n*)	% (*n*)	
Socioeconomic characteristics			
Mother's age (<30 years)	40.0 (205)	28.6 (154)	<0.001
Mother without a job at 6 months after birth	33.4 (173)	48.3 (259)	<0.001
≤first 3 years of secondary school (mother)	23.3 (120)	28.6 (154)	0.03
≤first 3 years of secondary school (father)	22.8 (110)	26.2 (134)	<0.005
Belgian native mother	71.8 (377)	28.2 (280)	<0.001
Household income (≤2000 euros)	37.7 (177)	52.5 (249)	<0.001
Infant's and birth characteristics			
First born infant	42.9 (225)	40.3 (218)	0.4
Male	51.8 (271)	51.6 (280)	0.9
Delivery in BFHI	23.5 (118)	68.7 (354)	<0.001
Caesarean	21.8 (114)	20.5 (110)	0.6
Preterm	7.8 (41)	5.3 (30)	0.13
MCH attendance	72.6 (382)	78.3 (426)	0.03
Breastfeeding			
Exclusive BF at birth	73.3 (385)	83.3 (453)	<0.001
Exclusive BF at discharge of the maternity unit	66.4 (348)	80.9 (440)	<0.001
Any BF at birth	81.7 (429)	93.0 (506)	<0.001
Any BF at discharge of the maternity unit	76.4 (400)	90.6 (493)	<0.001
Partially supplemented BF at discharge of the maternity unit	10.0 (44)	9.7 (53)	0.9
Median duration of exclusive BF (month)	3.0 (0.03–11.0)°	4.0 (0.03–11.0)°	<0.001
Median duration of any BF (month)	4.0 (0.03–24.0)°	6.0 (0.03–24.0)°	<0.001
Breastfeeding characteristics			
Intention of BF before pregnancy	65.2 (330)	72.8 (388)	0.008
Awareness of WHO recommendation	26.3 (138)	20.3 (111)	0.02
Negative partner's attitude	24.4 (122)	12.1 (63)	<0.005

°min–max.

**Table 3:** Distribution of desired duration (% (*n*), *P* value) and median (min–max) performed duration (months), 95% CI *P* value, according to desired duration.

	Desired duration			Achieved duration			
	Wallonia	Brussels	*P*	Wallonia	*P*	Brussels	*P*
	% (*n*)	% (*n*)		Median duration 95% CI		Median duration 95% CI	
<6 months	16.5 (58)	9.5 (33)	<0.001	1.5 (0.9–2.1)	<0.001	2.0 (0.8–3.2)	<0.001
6 months	20.8 (73)	15.9 (55)		3.0 (2.6–3.5)		4.0 (3.3–4.7)	
7–11 months	8.8 (31)	8.1 (28)		6.0 (4.9–7.1)		6.0 (3.4–8.7)	
≥12 months	21.9 (77)	53.3 (185)		6.0 (5.6–6.4)		7.0 (6.3–7.7)	
As long as the baby and myself wanted to	31.9 (112)	13.3 (46)		4.0 (3.1–5.0)		8.0 (5.4–10.6)	

**Table 4:** Mothers' dissatisfaction (%, *n*) based on sample characteristics among mothers who breastfed at birth in Wallonia and Brussels (OR, 95% CI, *P*).

	Wallonia			Brussels		
	% (*n*)	Crude OR (95% CI)	*P* value	% (*n*)	Crude OR (95% CI)	*P* value
Mother's age (years)						
<30	72.1 (106)	1.9 (1.2–3.0)	0.003	66.2 (90)	1.7 (1.1–2.6)	0.008
≥30	57.1 (140)	1		52.9 (181)	1	
Awareness of WHO recommendation						
Yes	46.5 (55)	1		42.6 (43)	1	
No	70.0 (198)	2.7 (1.7–4.2)	<0.001	60.5 (230)	2.1 (1.3–3.2)	<0.001
Mother's education level						
≤first 3 years of sec. school	68.9 (93)	1.8 (1.1–3.0)	0.01	61.8 (84)	1.6 (1.0–2.5)	0.05
Last 3 years of sec. school	70.5 (62)	2.0 (1.1–3.5)		61.7 (82)	1.6 (1.0–2.5)	
Higher education	54.9 (96)	1		50.5 (105)	1	
PIM						
Yes	75.3 (58)	2.0 (1.1–3.6)	0.01	68.9 (62)	1.9 (1.2–3.0)	0.01
No	60.0 (195)	1		54.0 (211)	1	
Preterm infant						
Yes	80.0 (24)	2.5 (1.0–6.2)	0.04	64.0 (16)	1.4 (0.6–3.2)	0.5
No	61.6 (229)	1		54.6 (257)	1	
Duration desired (month)			0.04			<0.001
<6	65.0 (39)	1.2 (0.6–2.4)		75.8 (25)	3.6 (1.2–10.9)	
6	77.8 (56)	2.2 (1.1–4.6)		73.6 (39)	3.2 (1.3–8.1)	
7–11	64.5 (20)	1.1 (0.5–2.8)		72.4 (21)	3.0 (1.0–10.0)	
≥12	79.2 (61)	2.4 (1.2–4.9)		87.6 (163)	8.1 (3.7–18.0)	
As long as the baby and I wanted to	61.4 (70)	1		46.7 (21)	1	
First born children						
Yes	69.5 (123)	1.7 (1.1–2.5)	0.01	57.8 (108)	1.1 (0.7–1.6)	0.7
No	57.6 (129)	1		55.8 (163)	1	
Father's education level						
≤first 3 years of sec. school	55.3 (96)	1.4 (0.9–2.4)	0.1	67.2 (82)	2.1 (1.3–3.5)	0.005
Last 3 years of sec. school	68.8 (55)	1.7 (0.9–3.1)		59.2 (84)	1.5 (1.0–2.4)	
Higher education	56.8 (83)	1		49.0 (94)	1	
Delivery in BFHI						
Yes	61.8 (55)	1		54.6 (172)	1	
No	65.0 (195)	1.1 (0.7–1.9)	0.6	63.8 (90)	1.5 (1.0–2.2)	0.07
Household income						
<2000 euros	68.2 (88)	1.3 (0.9–2.1)	0.2	61.7 (140)	1.4 (1.0–2.1)	0.06
>2000 euros	61.3 (141)	1		52.6 (103)	1	
Birth month						
May–September	60.6 (172)	1		53.6 (178)	1	
October-November	68.1 (79)	1.4 (0.9–2.2)	0.2	63.8 (95)	1.5 (1.0–2.2)	0.04
Infant's age when mother returned to work			0.05			1.0
≤3 months	72.5 (58)	1.9 (1.1–3.6)		56.4 (31)	1.0 (0.5–1.9)	
4 months and more	57.6 (110)	1		56.5 (108)	1	
Unemployed	65.6 (84)	1.4 (0.9–2.3)		56.7 (131)	1.0 (0.7–1.5)	

Never significant at level 0.05: maternity BF type, infant's sex, mode of delivery, prenatal maternal intention, partner's attitude, and mother's native nationality.

**Table 5:** Top 15 reasons for not breastfeeding and weaning in the first year (%).

	At the maternity ward		After the maternity ward (months)		
	No BF	Partial BF	≤3	≤6	≤12
Prevalence of any BF					
Wallonia (W)	23. 6	10.0	54.1	29.1	11.8
Brussels (B)	9.4	9.7	73.9	55.7	23.3
Reason of weaning					
*1/BF intrinsic problems *	10.4	**60.9 **	**58.5 **	38.4	31.5
	17.6	**63.5**	**64.9**	34.8	35.3
PIM					
W	6.5	**37.0**	**31.1**	**20.5**	**18.5**
B	5.9	**48.1**	**26.7**	**26.6**	**25.2**
Technical difficulties					
W	2.6	**23.9**	**15.2**	3.6	1.9
B	8.8	**15.4**	15.3	1.8	1.7
No milk					
W	1.3	—	12.2	14.3	11.1
B	2.9	—	**22.9**	6.4	8.4
*2/External causes *	**89.6**	39.1	41.5	**61.6**	**68.5**
	**82.4**	36.5	35.1	**65.2**	**64.7**
Personal opinion					
W	**37.7**	—	4.8	—	—
B	**32.4**	3.8	3.0	—	0.8
Back to work					
W	1.3	—	12.8	**41.1**	**25.9**
B	2.9	—	9.2	**26.6**	12.6
Mother's health					
W	**13.0**	4.3	7.3	6.3	9.3
B	5.9	3.8	6.1	7.3	4.2
Logistics					
W	7.8	—	1.8	1.8	3.7
B	2.9	—	3.8	3.7	5.9
Twins					
W	1.3	10.9	3.7	0.9	—
B	4.3	13.5	4.6	—	—
Fatigue					
W	—	—	7.3	3.6	11.1
B	—	—	4.6	7.3	5.0
The child did not want to drink					
W	—	2.2	4.3	7.1	11.1
B	—	1.9	5.3	11.0	**23.5**
Circum. related to childbirth					
W	5.6	10.9	—	—	—
B	8.7	5.8	—	—	—
Previous bad experience					
W	**13.0**	8.7	—	—	—
B	5.9	—	—	—	—
Baby's health					
W	2.6	8.7	—	—	—
B	8.7	3.8	—	—	—
Pregnant mother					
W	—	—	—	—	5.6
B	—	—	—	2.8	10.9
Other reasons					
W	3.3	0	1.2	0.9	7.4
B	10.9	1.9	3.8	3.7	1.7
*N* total	77	46	164	112	54
	34	52	131	109	119

**Table 6:** PIM according to the sample characteristics among the mothers who were breastfeeding when leaving the ward for at least 6 months (any type of BF) and among those who were breastfeeding exclusively for less than 5 months (% (*n*), *P* values).

	Weaning exclusive BF before 5 months		Weaning any BF before 6 months	
	Wallonia	Brussels	Wallonia	Brussels
BF prevalence at 5 and 6 months	17.7%	38.4%	29.1%	55.7%
Proportion of PIM	23.8 (68/286)	20.7 (70/338)	23.8 (68/286)	25.0 (47/188)
Predictors				
BF at maternity ward				
Exclusive	—	—	25.7 (61)	22.4 (35)
Partial			23.5 (8)	37.9 (11)
*P* value			0.8	0.08
Mother's education level				
≤first 3 years of sec. school	29.5 (31)	25.8 (24)	26.9 (28)	37.5 (18)
Last 3 years of sec. school	34.4 (21)	19.4 (18)	39.2 (20)	20.0 (11)
Higher education	12.9 (15)	16.8 (25)	17.7 (20)	20.2 (17)
*P* value	**0.001**	0.3	**0.01**	0.05
Father's education level				
≤first 3 years of sec. school	21.5 (23)	28.9 (24)	20.8 (21)	31.3 (15)
Last 3 years of sec. school	37.1 (23)	18.2 (16)	40.4 (23)	28.3 (13)
Higher education	17.5 (17)	17.4 (25)	21.7 (20)	19.5 (16)
*P* value	**0.01**	0.09	**0.01**	0.3
Awareness of the WHO recommendations				
Yes	12.5 (9)	22.6 (14)	13.8 (8)	21.1 (8)
No	27.6 (59)	20.3 (56)	28.6 (61)	25.8 (42)
*P* value	**0.009**	0.7	**0.02**	0.6
Mother delivery in BFHI				
Yes	32.4 (23)	21.4 (46)	32.4 (23)	22.4 (35)
No	21.4 (45)	19.0 (20)	21.4 (45)	37.9 (11)
*P* value	0.06	0.6	0.06	0.08
Preterm				
Yes	53.3 (8)	20.5 (65)	53.3 (8)	24.9 (44)
No	22.1 (60)	23.5 (5)	22.1 (60)	27.3 (3)
*P* value	**0.006**	0.7	**0.006**	1.0

Never significant at level 0.05: birth season, attending a MCH, BHFI, infant's sex, and mother's native nationality.
